# Movement Patterns and Match Statistics in the National Rugby League Women's (NRLW) Premiership

**DOI:** 10.3389/fspor.2021.618913

**Published:** 2021-02-11

**Authors:** Tim Newans, Phillip Bellinger, Simon Buxton, Karlee Quinn, Clare Minahan

**Affiliations:** ^1^Griffith Sports Science, Griffith University, Gold Coast, QLD, Australia; ^2^Queensland Academy of Sport, Nathan, QLD, Australia; ^3^National Rugby League, Sydney, NSW, Australia

**Keywords:** female, load monitoring, external output, mixed model, match demands, GPS

## Abstract

As women's rugby league grows, the need for understanding the movement patterns of the sport is essential for coaches and sports scientists. The aims of the present study were to quantify the position-specific demographics, technical match statistics, and movement patterns of the National Rugby League Women's (NRLW) Premiership and to identify whether there was a change in the intensity of play as a function of game time played. A retrospective observational study was conducted utilizing global positioning system, demographic, and match statistics collected from 117 players from all NRLW clubs across the full 2018 and 2019 seasons and were compared between the ten positions using generalized linear mixed models. The GPS data were separated into absolute (i.e., total distance, high-speed running distance, and acceleration load) and relative movement patterns (i.e., mean speed, mean high speed (> 12 km·h^−1^), and mean acceleration). For absolute external outputs, fullbacks covered the greatest distance (5,504 m), greatest high-speed distance (1,081 m), and most ball-carry meters (97 m), while five-eighths recorded the greatest acceleration load (1,697 m·s^−2^). For relative external outputs, there were no significant differences in mean speed and mean high speed between positions, while mean acceleration only significantly differed between wingers and interchanges. Only interchange players significantly decreased in mean speed as their number of minutes played increased. By understanding the load of NRLW matches, coaches, high-performance staff, and players can better prepare as the NRLW Premiership expands. These movement patterns and match statistics of NRLW matches can lay the foundation for future research as women's rugby league expands. Similarly, coaches, high-performance staff, and players can also refine conditioning practices with a greater understanding of the external output of NRLW players.

## Introduction

The National Rugby League Women's (NRLW) Premiership is the highest level of domestic rugby league competition for women in Australia. The NRLW was introduced in 2018 involving a three-round competition between four teams, culminating in a grand final between the two highest-ranked teams. The NRLW follows the international rugby league rules set by the Rugby League International Federation with the exception that NRLW matches are 60 min comprised of two 30-min halves, half time is 15 min, allow ten interchanges in each match, and observe a 40/30 kick advantage.

Describing the movement patterns of a given sport lays the foundations for future research to examine the intricacies of match play. While female soccer movement patterns have been established as seen in a recent review (Griffin et al., 2020), Clarke et al. have articulated the movement patterns in both Australian-rules football (Clarke et al., 2018, 2019) and rugby sevens (Clarke et al., 2015, 2017). Similarly, a review of movement patterns in field-based sports spanning maximal speed, high-speed thresholds and movement patterns has been conducted in women (Hodun et al., 2016); however, the movement patterns of players competing in elite-level women's rugby league has been rarely explored in the scientific literature (Quinn et al., 2020). With an 18% year-on-year growth in women's rugby league participation (National Rugby League, 2020), the NRLW Premiership is set to expand in the number of teams over the coming years. To ensure that the level of competition does not regress as a function of including more teams, there is a need for emerging players to be sufficiently trained and conditioned to compete at the elite level, such as those described in the present study.

The movements of players in the Australian women's rugby league team during international match-play have previously been recorded using global positioning system (GPS) technology (Quinn et al., 2020). The findings of this initial study reported that players covered 6,500 m in total distance, with ~500 m covered above 15 km·h^−1^. This study also displayed the relative change in the intensity of match play across each half, whereby mean high speed (>12 km·h^−1^) was reduced by ~40% from the start to the end of the first half (Quinn et al., 2020). While backs were shown to exhibit greater absolute distance covered, there were no significant differences in the relative distances between forwards and backs. In female soccer, it was shown that domestic movement patterns were significantly lower than in international-level matches (Andersson et al., 2010); however, it remains to be seen whether this is present in rugby league. As there is a disparity in match duration from the NRLW Premiership to international matches and as there is varying quality of opposition in women's international rugby league (Quinn et al., 2020) which can affect movement patterns (Hulin et al., 2015), the proportional change in movement patterns from domestic to international rugby league may not imitate the change seen in soccer (Andersson et al., 2010).

There is no doubt that the study by Quinn et al. (2020) provides important information regarding the movement patterns of elite, international rugby league players. However, a common critique in the literature when describing player movement patterns is that studies typically only gather data from one or two teams (Glassbrook et al., 2019). By gathering data from only a few teams, team tactics and strategy could obscure the true movement patterns inherent across the entire competition (Glassbrook et al., 2019). As the only previous investigation in women's rugby league was in international rugby league and focused only on a single team (Quinn et al., 2020), this study will examine the GPS data from the four domestic NRLW teams during the 2018 and 2019 seasons to describe the movement patterns of elite, domestic women's rugby league players. Similarly, in studies with only one or two teams, there is usually only enough power to segregate players into three positional groups; however, there have now been studies in men's rugby league that have segregated players into more specific positional groups (Delaney et al., 2015, 2016a). Therefore, in the present study, we expand the comparisons to further quantify the effect of playing position on the match statistics (tackles, runs, etc.) and GPS metrics (distance, velocity, acceleration).

## Materials and Methods

The present study included 117 players from the four NRLW clubs (age = 26.8 ± 5.4 yr; height = 1.68 ± 0.07 m; body mass = 76.7 ± 11.9 kg). The Griffith University Human Ethics Committee approved this study (GU Ref No: 2019/359). There were 475 match entries as one player did not enter the field during one match. The mean number of matches played by each player was 4.1 ± 2.2 matches.

All match statistics and demographics (i.e., age, height, and body mass) were publicly sourced from NRL.com. The definitions of match statistics were determined by STATS, the National Rugby League's statistics provider. “All runs” were defined as any time the ball carrier went into contact with a defender, “all run metres” was the cumulative distance of all runs, “tackles” were when the defender successfully executed a tackle, “missed tackles” were when the defensive player could not bring the attacking player to the ground or successfully complete the tackle, and “tackle breaks” were when the attacking player was able to continue running after a missed tackle. These five statistics were chosen by the authors to reflect the contact nature of rugby league and, therefore, to highlight the relative position differences in these events.

The movement patterns of all players during NRLW matches were collected using 10 Hz Optimeye S5 GPS units (Catapult Sports, Victoria, Australia). The GPS data were routinely collected at each match by the sport scientists at each club, while the National Rugby League oversaw the collection, amalgamation and provision of the datasets to the authors in “.cpf” form. Of the 475 match entries that had match statistics recorded, 370 were available for GPS analysis. This discrepancy was due to several reasons, with poor satellite coverage within the stadium being the main reason for why files were not provided to the authors for analysis. Regardless of whether an athlete's GPS data was available, the match statistics were still included in the analysis. Match files were segmented into halves within the proprietary software and interchanges were recorded in the software as per the match footage and GPS tracing. Velocity zones (V1-6) were aligned with those previously reported in women's rugby league (Quinn et al., 2020), with V1 set at 0–6 km·h^−1^, V2 set at 6.01–9 km·h^−1^, V3 set at 9.01–12 km·h^−1^, V4 set at 12.01–15 km·h^−1^, V5 set at 15.01–18 km·h^−1^, and V6 set at >18 km·h^−1^. Acceleration load was calculated as the summation of absolute acceleration and deceleration values across the duration of the match. Three absolute output metrics [total distance, distance >12 km·h^−1^ (i.e., high-speed running; HSR), and acceleration load (Delaney et al., 2016a)] were divided by the total time spent on the field to calculate the mean speed (MS), mean speed when traveling >12 km·h^−1^ (MS_12_), and mean acceleration (Andersson et al., 2010), respectively, to represent the relative outputs. The term “absolute” was used to describe the sum of the metric throughout a match, while the term “relative” was used when dividing the absolute metric by time. Twelve kilometers per hour was used as the threshold for high-speed running to draw comparisons to the previous women's rugby league literature (Quinn et al., 2020).

To assess the positional differences for each tactical match statistic, GPS data metric, and the age of each position, GLMM's were employed, with the associated R script is attached as Appendix 1. All GLMM's were built using the *lme4* (Bates et al., 2015) package in R version 3.5.2 (R Core Team, 2019), the *afex* package (Singmann et al., 2020) was used to determine significance at α = 0.05 for all analyses, the *emmeans* package (Lenth, 2020) was used for pairwise comparisons, while the *sjPlot* package (Lüdecke, 2020) was used for model diagnostics. The dataset was arranged in “long form” with each observation for each player on a new row. After loading in the *lme4* and *afex* packages, the first model was built as seen in line 16. For each model, the dependent variable was the metric we were interested in explaining (“age,” “tackles made,” “total distance,” etc.), a fixed effect of position was inserted, with random effects of “player,” “match,” and “team” inserted as well to remove the variability attributed to these variables. Finally, the shape of the distribution was identified for each model with most models following a Gaussian distribution except those with count data which followed a Poisson distribution.

Once the full model (i.e., all fixed and random effects included, see Line 16 in Appendix 1) was established for each dependent variable, each random effect was consecutively removed to identify which random effects were required in the model. If the Bayesian Information Criterion (BIC) was lower after removing a random effect (see Line 18 in Appendix 1) and an analysis of variance between the full and the reduced model was significant (see Line 19 in Appendix 1), it was deemed a significant contributor to the model, otherwise it would be subsequently dropped from that model. Once only random effects that significantly contributed to the model were remaining, the reduced model was deemed the most parsimonious. In all models, the match ID was deemed a significant random effect, while the team that each player competed for was not deemed a significant random effect in any of the models. Once the reduced model was identified, the null model was established (i.e., the reduced model without the predictor included, see Line 20 in Appendix 1). If the calculated X^2^ statistic from an analysis of variance between the reduced model and the null model was significant (*p* < 0.05), then the predictor variable was deemed to significantly contribute to the model, To check the assumptions, the reduced model's residuals were plotted against its fitted value to determine if there were any patterns emerging (see Line 22 in Appendix 1) (Harrison et al., 2018).

GLMM's were also used to determine whether the duration of play affected the MS (i.e., players who play shorter duration perform at a higher intensity). Due to the nature of interchange in rugby league, some positions were more likely to play the full 60 min than other positions. Consequently, 93% of backs (i.e., fullbacks, wingers, and centers), 80% of halves (i.e., five-eighths and halfbacks), 59% of second-rowers, 46% of hookers, 21% of locks, and 4% of props played the full duration of the match. If the regression coefficient calculated was significantly different (*p* < 0.05) from zero (see Line 42 of Appendix 1), it displayed a relationship between the amount of time played and the relative output of the position. For the positions where there was a significant effect of duration on MS, the slopes were reported alongside the MS.

Finally, GLMM's were used to determine the change in output in different velocity bands across the two 30-min halves. Like the positional analysis, a full model (see Line 63 in Appendix 1) was developed, with non-significant random effects removed in the reduced model (see Line 65 in Appendix 1). An analysis of variance was performed between the reduced model and the null model (i.e., the reduced model with “half” excluded) to calculate a X^2^ statistic to determine whether there was a significant difference (*p* < 0.05) in external output measures between the two halves.

## Results

The demographics and minutes played across each playing position are displayed in Table 1. Second-row players were the tallest, while locks and hookers were the shortest. Props were considerably heavier than all other positions, with fullbacks and hookers the lightest of the positions. There was only one significant difference in age between the positions [X^2^_(9)_ = 17.59, *p* = 0.040], with hookers significantly older than fullbacks.

**Table 1:** Demographics and technical data of NRLW players by position.

**Position**	**Unique players (*n*)**	**Match entries (*n*)**	**Height (cm)**	**Body mass (kg)**	**Age (yr)**	**Game time (min)**	**All run meters (m)**	**All runs (*n*)**	**Tackles (*n*)**	**Missed tackles (*n*)**	**Tackle breaks (*n*)**
Fullback	8	28	168.4 ± 4.2	66.8 ± 5.2	26.1(24.9–27.3)	60(60–60)	97.2(82.0–112.4)	10.3(8.5–12.5)	4.8(1.9–7.7)	1.3(0.9–2.0)	3.5(2.4–4.7)
Winger	19	56	168.0 ± 5.5	69.4 ± 5.0	26.6(25.4–27.7)	60(60–60)	66.4^a^(55.9–76.9)	7.5(6.5–8.8)	4.1(2.1–6.1)	1.3(0.9–1.7)	3.1(2.2–3.9)
Center	17	56	168.3 ± 6.9	71.4 ± 4.4	26.2(25.1–27.3)	60(60–60)	82.9(72.1–93.7)	9.4(8.2–10.8)	9.4^b^(7.3–11.5)	2.0(1.6–2.6)	3.0(2.2–3.8)
Five-Eighth	10	28	168.1 ± 6.4	70.9 ± 7.8	27.2(26.2–28.3)	60(60–60)	38.6^a, c^(24.4–52.8)	5.2^a, c^(4.1 – 6.6)	14.3^a, b^(11.6–16.9)	2.5(1.8–3.5)	0.9^a^(−0.2 to 2.0)
Halfback	8	28	168.7 ± 7.1	70.8 ± 11.4	26.6(25.5–27.7)	60(59–60)	44.8^a, c^(29.9–59.8)	6.3^a^(5.1–7.8)	12.5^a, b^(9.6–15.3)	3.0^b^(2.2–4.0)	1.5(0.4–2.5)
Hooker	6	28	164.5 ± 6.5	67.5 ± 7.0	27.6(26.5–28.7)	55(41–60)	42.9^a, c^(27.3–58.4)	5.3^a, c^(4.1–6.9)	26.4^a, b, c, d, e^(23.5–29.4)	3.4^a, b^(2.5–4.8)	1.1(-0.2–2.4)
Prop	23	56	171.1 ± 6.2	90.6 ± 8.5	27.0(26.0–28.0)	35(30–39)	69.8^a, d^(59.9–79.7)	7.4(6.4–8.6)	16.1^a, b, c, f^(14.3–17.9)	1.6^f^(1.2–2.1)	1.1^a, b, c^(0.4–1.9)
Second-Row	15	56	171.6 ± 6.2	81.5 ± 6.8	26.8(25.8–27.9)	60(53–60)	65.1^a^(54.6–75.6)	8.0(6.9 – 9.3)	17.8^a, b, c, f^(15.8–19.8)	2.6^b^(2.0–3.2)	2.6(1.8–3.3)
Lock	9	28	164.4 ± 8.2	75.4 ± 4.8	27.0(26.0–28.1)	44(37–55)	53.2^a, c^(39.0–67.5)	7.2(5.9–8.9)	22.9^a, b, c, d, e, g, h^(20.3–25.5)	3.2^a, b, g^(2.4–4.4)	2.3(1.2 – 3.4)
Interchange	48	111	167.5 ± 7.1	80.2 ± 13.4	26.9(25.9–28.0)	24(18–33)	47.7^a, c, g^(40.7–54.6)	5.5^a, b, c, g, h^(4.9–6.2)	11.9^a, b, f, g, h, i^(10.6–13.2)	1.6^e, f, i^(1.3–2.0)	1.7(1.2–2.3)

*N.B. All values are displayed as mean (95% CI) except height and body mass are displayed as mean ± SD and game time which is median (IQR), superscript indicates significantly different from ^a^fullback, ^b^winger, ^c^centre, ^d^five-eighth, ^e^halfback, ^f^hooker, ^g^prop, ^h^second-row, ^i^lock (p < 0.05)*.

Table 1 also displays the tactical match statistics for each playing position. Most of the backs, halves, and second-row players completed the full 60 min of match play. Fullback players recorded the most runs [X^2^_(9)_ = 50.64, *p* < 0.001], run meters [X^2^_(9)_ = 49.96, *p* < 0.001], and tackle breaks [X^2^_(9)_ = 32.81, *p* < 0.001], while hookers recorded the most tackles [X^2^_(9)_ = 153.02, *p* < 0.001].

As most fullback, wing, center, five-eighth, and halfback players completed the full 60 min, it was not necessary to determine the coefficient for the relationship between MS and minutes played. This relationship was non-significant for hooker, prop, second-row and lock players; however, it was significant for interchange players [X^2^_(1)_ = 24.41, *p* < 0.001] and therefore needed to be accounted for when calculating MS. For relative outputs, there were no significant differences in MS [X^2^_(9)_ = 14.61, *p* = 0.102] and MS_12_ [X^2^_(9)_ = 8.04, *p* = 0.530], between any of the positions, while mean acceleration [X^2^_(9)_ = 21.26, *p* = 0.012] was significantly different between wingers and interchange. For absolute output, total distance [X^2^_(9)_ = 195.41, *p* < 0.001], HSR [X^2^_(9)_ = 115.16, *p* < 0.001], and acceleration load [X^2^_(9)_ = 183.14, *p* < 0.001] were significantly different between positions (Table 2). When comparing the first and second halves, there were no significant differences in the relative distances covered in any of the speed zones, as well as the overall MS. The parameter estimates can be seen in Table 3.

**Table 2:** External workload in the NRLW by player position.

**Position**	**Mean speed by minutes**	**Mean speed (m·min^**−1**^)**	**Mean high speed (m·min^**−1**^)**	**Total distance (m)**	**High-speed running (>12 km·h^**−1**^) (m)**	**Acceleration load (m·s^**−2**^)**	**Mean acceleration (m·s^**−3**^)**
Fullback	–	80.1 (75.4–84.8)	15.3(12.1–18.6)	5,504(5,008–6,000)	1,081(915–1,246)	1,530(1,374–1,686)	0.37(0.34–0.40)
Winger	–	75.5(71.8–79.3)	13.1(10.4–15.8)	5,134(4,763–5,504)	916(780–1,052)	1,549(1,434–1,664)	0.38(0.35–0.40)
Center	–	75.9(72.3–79.6)	13.5(10.9–16.2)	5,116(4,760–5,473)	882(749–1,016)	1,582(1,472–1,693)	0.39(0.37–0.42)
Five-Eighth	–	79.8(75.3–84.3)	13.3(10.3–16.3)	5,244(4,754–5,733)	883(725–1,042)	1,697(1,545–1,850)	0.42(0.39–0.45)
Halfback	–	80.5(76.1–84.9)	14.3(11.4–17.2)	5,212(4,741–5,683)	900(745–1,055)	1,593(1,446–1,740)	0.41(0.39–0.44)
Hooker	−0.18(−0.41 to 0.12)	82.7(77.5–87.9)	15.2(11.7–18.7)	4,844.5(4,289–5,400)	817(636–999)	1,472(1,296–1,648)	0.42(0.39–0.45)
Prop	−0.11(−0.37 to 0.16)	78.6(75.1–82.1)	13.4(11.0–15.8)	2,908^a, b, c, d, e, f^(2,557–3,259)	432^a, b, c, d, e, f^(305–559)	887^a, b, c, d, e, f^(779–994)	0.41(0.39–0.43)
Second-Row	−0.12(−0.31 to 0.06)	78.0(74.3–81.6)	12.7(10.2–15.1)	4,627^g^(4,271–4,984)	693^a, b, g^(563–823)	1,362^d, g^(1,252–1,472)	0.39(0.37–0.41)
Lock	−0.22(−0.53 to 0.16)	78.4(74.0–82.9)	13.9(11.1–16.8)	4,044^a, b, c, d, e, g^(3,562–4,526)	697^a, g^(543–852)	1,228^b, c, d, e, g^(1,078–1,378)	0.40(0.38–0.43)
Interchange	−0.41*(−0.57 to −0.26)	80.1(77.0–83.1)	14.5(12.4–16.7)	2,514^a, b, c, d, e, f, h, i^(2,246–2,782)	439^a, b, c, d, e, f, h, i^(327–551)	775^a, b, c, d, e, f, h, i^ (695–855)	0.41^b^ (0.40–0.43)

N.B. All figures are presented as mean (95% CI), –indicates insufficient data to determine regression coefficient, ^*^ indicates sig. different from zero, superscript indicates significantly different from ^a^fullback, ^b^winger, ^c^centre, ^d^five-eighth, ^e^halfback, ^f^hooker, ^g^prop, ^h^second-row, ^i^lock (p < 0.05).

**Table 3:** Half-by-half analysis of external outputs obtained during domestic women's rugby league matches.

**Metric**	**1st Half**	**2nd Half**
Relative total distance (m·min^−1^)	79.6 (76.8–82.5)	79.3 (76.5–82.2)
Relative V1 distance (m·min^−1^)	36.7 (33.0–40.4)	36.9 (33.2–40.7)
Relative V2 distance (m·min^−1^)	15.1 (14.4–15.8)	15.0 (14.2–15.7)
Relative V3 distance (m·min^−1^)	14.5 (12.8–16.1)	14.1 (12.5–15.8)
Relative V4 distance (m·min^−1^)	7.7 (4.3–14.1)	7.6 (4.2–13.8)
Relative V5 distance (m·min^−1^)	2.9 (1.6–5.3)	2.9 (1.6–5.2)
Relative V6 distance (m·min^−1^)	2.0 (1.1–3.7)	1.8 (1.0–3.3)

*N.B. Values presented as mean (95% CI). V1 = 0–6 km·h^−1^, V2 = 6.01–9 km·h^−1^, V3 = 9.01–12 km·h^−1^, V4 = 12.01–15 km·h^−1^, V5 = 15.01–18 km·h^−1^, and V6 = >18 km·h^−1^. No significant differences (p < 0.05) were found between halves for any metric*.

## Discussion

The present study describes the absolute and relative (to time) movement patterns, player demographics, and match statistics of the 2018–2019 NRLW Premiership in Australia. With respect to differing absolute movement patterns, backs covered between 5,100 and 5,500 m with between 850 and 1,100 m above 12 km·h^−1^, five-eighths and halfbacks covered ~5,200 m with 900 m above 12 km·h^−1^, while forwards covered between 2,900 and 4,900 m with between 430 and 820 m above 12 km·h^−1^. Similar patterns were reflected in acceleration load where there were no significant differences between any back or halves position, while props were significantly lower than all other starting positions. However, when comparing movement patterns expressed relative to time, there were no significant differences in the relative distance metrics (MS and MS_12_) between any of the positions. The only significant difference in relative movement patterns was mean acceleration between wingers and interchange players. While the absolute movement patterns display a disparity in the typical match profile across positions of elite women's rugby league, the lack of difference in movement patterns relative to time means when designing training and conditioning protocols the intensity of play is matched across all positions and varying levels of total work is required between positions.

When assessing whether the MS changed as a function of the time on field, there were also no significant relationships for any of the starting positions, with a decrease of 0.4 m·min^−1^ for each minute played in the interchange players. While this decrease may seem insignificant practically, when comparing an interchange player competing for 10 min compared to 35 min, it equates to a decrease of 10 m·min^−1^, which is ~13% decrease in MS. These results reflect a similar pattern seen in the men's game where transient fatigue was attributed to the decline in intensity as an interchange player's bout was prolonged (Waldron et al., 2013). These results also display a need to further understand the role and requirement of interchange players in women's rugby league, similar to those explored in the men's game (Delaney et al., 2016b).

When comparing relative movement patterns between domestic (i.e., the present study) and international women's rugby league (Quinn et al., 2020) NRLW backs covered between 75.5 and 80.1 m·min^−1^ compared to ~75 m·min^−1^ in international matches, NRLW halves covered between 79.8 and 80.5 m·min^−1^ compared to ~78 m·min^−1^ in international matches, and NRLW forwards covered between 78.0 and 82.7 m·min^−1^ compared to ~71 m·min^−1^ in international matches. This contradicts figures seen in other codes of football played by women, with female soccer players recording a higher intensity when playing at the international level compared to at the domestic level (Andersson et al., 2010). These comparisons show that while the backs and halves roughly display similar MS, it is evident that forwards in the shorter-duration NRLW may maintain their output better than in the longer-duration international level. Conversely, it could be that the international players did not require as high output as they were of a higher quality than their opponents (Hulin et al., 2015).

While the values for MS and MS_12_ for the full match were comparable to Quinn et al. (Quinn et al., 2020) (who also found no significant differences in the 80-min players between any of the positions), when comparing half-to-half (Table 3), Quinn et al. (2020) found a significant decline in certain velocity bands from the first to second half, where the present study showed no such difference. This could be due to the differing inclusion criteria where Quinn et al. (2020) only included those playing the full 80-min in the half-to-half comparison. The MS in the present study was still lower than those seen in other sports played by women (Hodun et al., 2016), which could be attributed to the energetic demand of the tackling nature of rugby league which is not present in sports such as soccer and hockey. Although, the MS was higher than that recorded in women's rugby union (Suarez-Arrones et al., 2014) which would feasibly be the most similar sport code, which reflects the difference in movement patterns between men's rugby league (Cummins et al., 2018) and rugby union (Jones et al., 2015). However, due to the low threshold for mean high speed (i.e., 12 km·h^−1^), it is difficult to compare high-intensity efforts to other sports (Hodun et al., 2016). While the mean high-speed threshold was chosen to make direct comparisons with international women's rugby league, further investigations could use higher intensity thresholds to further interrogate the differences in movement patterns with other sports.

Another noteworthy finding was the unique characteristics of the lock position. While locks within modern men's rugby league are most commonly paired with second-rowers (i.e., the back row) or with props (i.e., the “middle forwards”) (Glassbrook et al., 2019), the present study showed that locks in NRLW were different in their role. NRLW locks were, on average, 8 and 7 cm shorter than second-rowers and props, respectively, weighed 6.1 and 15.2 kg less, respectively, ran for 11.9 and 16.8 m less with the ball, respectively, and made 5.1 and 6.8 more tackles, respectively. Consequently, these findings reinforce the limitations in simply transferring male rugby league conditioning research into the female game (Emmonds et al., 2019).

The data from the present study is crucial as it is the first week-by-week examination of women's rugby league. Quinn et al. (Quinn et al., 2020) provided an overview of the movement patterns of the Australian Women's Rugby League team during international competition (Quinn et al., 2020); however, these movement patterns could be adversely affected by cumulative fatigue, as previously seen in male rugby league (Johnston et al., 2013). As the NRLW Premiership expands, it will conceivably shift from a semi-professional to a professional competition enabling players to commit full-time to their career in rugby league. This shift, with players unconstrained by holding secular jobs, may also see the movement patterns of NRLW match play increase above the values seen in this study.

Another notable feature of the present study was the dissemination of knowledge, skills, and awareness around the use of the mixed models for sports science practitioners. As noted in a recent call to sports scientists (Sainani et al., 2020), there is a growing need for sports scientists to be familiar with statistical methods that can properly account for variation within their datasets. While the requirement to account for repeated measures and missing data is pertinent in longitudinal GPS movement pattern studies, it is still lacking within a substantial proportion of the sports science community. For instance, in a recent systematic review of rugby league (Dalton-Barron et al., 2020), only seven of the 15 studies correctly accounted for the repeated observations within GPS datasets. Throughout this study, the authors have highlighted the importance of using appropriate statistical modeling for repeated measures of individuals and teams in longitudinal datasets. We explained the methodology and application of a GLMM as well as provide the relevant computer scripts required to perform a GLMM. We anticipate that this approach will increase the transparency and reproducibility of the findings, as well as encourage statistical literacy for sports scientists.

## Conclusion

This study was the first study to describe the movement patterns and match statistics in domestic elite-level women's rugby league. Two seasons of the NRLW Premiership (13 matches) were analyzed, with all clubs contributing to the dataset. While backs and halves covered significantly greater absolute distances than forwards, the relative distances covered were not different across any of the positions. The lock position was also a substantially different position to men's rugby league. This study also provided more clarity on the application of mixed models for sports science datasets, with a specific focus on team-sport data with repeated observations.

## Data Availability Statement

The raw data supporting the conclusions of this article will be made available by the authors, without undue reservation.

## Ethics Statement

The studies involving human participants were reviewed and approved by Griffith University Human Research Ethics Committee. Written informed consent for participation was not required for this study in accordance with the national legislation and the institutional requirements.

## Author Contributions

TN: project concept, data collection and analysis, and preparation of manuscript. PB and CM: project concept, refining, and synthesizing manuscript. KQ and SB: refining and synthesizing manuscript. All authors contributed to the article and approved the submitted version.

## Conflict of Interest

The authors declare that the research was conducted in the absence of any commercial or financial relationships that could be construed as a potential conflict of interest.
