# Supplementary material for: Movement Patterns and Match Statistics in the National Rugby League Women's (NRLW) Premiership
Source: Front Sports Act Living. 2021 Feb 11;3:618913. doi: 10.3389/fspor.2021.618913 (PMC7904888; doi:10.3389/fspor.2021.618913)
Supplement: Supplementary file 1 [file Table_1.DOCX]

library(afex)

library(effects)

library(ggplot2)

library(emmeans)

library(chron)

library(dplyr)

library(sjPlot)

NRLW <- read.csv("NRLW_GPS_Tactical.csv")

options(scipen = 999)

NRLW$FieldTime <- chron(times = as.character(NRLW$FieldTime))

NRLW$BenchTime <- chron(times = as.character(NRLW$BenchTime))

NRLW$MeanHSR <- NRLW$HSR / NRLW$FieldTime / 24 / 60

## Positional Differences in Tactical and GPS Data

full_model <- glmer("Variable" ~ Position + (1| Team) + (1 | Match_ID) + (1 | Player), data = NRLW, family = "gaussian") # Build first model

summary(full_model) # Inspect fixed and random effects

reduced_model <- glmer("Variable" ~ Position + (1 | Match_ID) + (1 | Player), data = NRLW, family = "gaussian") # Build second model without non-significant random effects

anova(full_model, reduced_model) # Determine if model is significantly affected

null_model <- glmer("Variable" ~ (1 | Match_ID) + (1 | Player), data = NRLW, family = "gaussian") # Build null model to determine if position is a significant contributor

anova(reduced_model, null_model) # Determine if model is significantly affected

plot_model(reduced_model, type = "diag") # Assess model diagnostics

Position_Estimates <- as.data.frame(effect("Position", full_model)) # Create parameter estimates based on position

Position_Estimates %>%

select(Position, fit, lower, upper) %>%

mutate_at(vars(fit, lower, upper), round, 1) # Clean dataframe for reporting

Pairwise_Positions <- as.data.frame(pairs(emmeans(full_model, "Position"))) # Create pairwise comparisons between positions

Pairwise_Positions %>%

filter(p.value < 0.05) # Determine significant pairwise comparisons

### Movement patterns as a function of minutes played

ggplot(data = NRLW[NRLW$Position == "Interchange", ], aes(x = Minutes, y = AveSpeed)) +

geom_point() +

geom_smooth(method = "lm") # Visualise the data

full_model <- glmer("Variable" ~ Minutes + (1 | Match_ID) + (1 | Player), data = NRLW, family = "gaussian") # Build first model

summary(full_model) # Inspect fixed and random effects

null_model <- glmer("Variable" ~ (1 | Match_ID) + (1 | Player), data = NRLW, family = "gaussian") # Build null model to assess if minutes is a significant contributor

anova(full_model, null_model) # Determine if model is significantly affected

plot_model(full_model, type = "diag") # Assess model diagnostics

## Half-to-half comparison of movement patterns

NRLW_Halves <- read.csv("NRLW_GPS_Tactical_Halves.csv") # Load in half-by-half data

NRLW_Halves <- NRLW_Halves %>%

mutate(

AveSpeed = TotalDistance / (FieldTime * 1440),

V1Speed = V1Distance / (FieldTime * 1440),

V2Speed = V2Distance / (FieldTime * 1440),

V3Speed = V3Distance / (FieldTime * 1440),

V4Speed = V4Distance / (FieldTime * 1440),

V5Speed = V5Distance / (FieldTime * 1440),

V6Speed = V6Distance / (FieldTime * 1440),

MeanHSR = HSR / (FieldTime * 1440),

MeanAccel = Acceleration.Load / (FieldTime * 86400)

) # Create new speed metrics

full_model <- glmer("Variable" ~ Half + (1 | Team) + (1 | Match_ID) + (1 | Player), data = NRLW_Halves, family = "gaussian") # Build first model

summary(full_model) # Inspect fixed and random effects

reduced_model <- glmer("Variable" ~ Half + (1 | Match_ID) + (1 | Player), data = NRLW_Halves, family = "gaussian") # Build second model without non-significant random effects

anova(full_model, reduced_model) # Determine if model is significantly affected

reduced_model <- glmer("Variable" ~ (1 | Match_ID) + (1 | Player), data = NRLW_Halves, family = "gaussian") # Build null model without half

anova(reduced_model, null_model) # Determine if model is significantly affected

plot_model(reduced_model, type = "diag") # Assess model diagnostics

Half_Estimates <- as.data.frame(effect("Half", full_model)) # Create parameter estimates based on half

Half_Estimates %>%

select(Position, fit, lower, upper) %>%

mutate_at(vars(fit, lower, upper), round, 1) # Clean dataframe for reporting
